# PREF-NET: a patient preference and experience study of lanreotide autogel administered in the home versus hospital setting among patients with gastroenteropancreatic neuroendocrine tumours in the UK

**DOI:** 10.1007/s00520-024-08377-7

**Published:** 2024-02-29

**Authors:** Mohid S. Khan, Kathryn Cook, Martin O. Weickert, Louise Davies, D. Mark Pritchard, Melissa Day, Tahir Shah, Diana Hull, Martyn Caplin, Melissa Back, Christelle Pommie, Kate Higgs

**Affiliations:** 1 Cardiff and Vale University Health Board, Cardiff, UK; 2 The ARDEN NET Centre, ENETS Centre of Excellence, University Hospitals Coventry & Warwickshire NHS Trust, Coventry, UK; 3 Liverpool University Hospitals NHS Foundation Trust, Liverpool, UK; 4 University Hospitals Birmingham NHS Foundation Trust, Birmingham, UK; 5 Royal Free London NHS Foundation Trust, London, UK; 6 Ipsen, Boulogne-Billancourt, France; 7 Ipsen, Slough, UK

**Keywords:** Homecare, Patient preference, Somatostatin analogue, Gastroenteropancreatic neuroendocrine tumours, Lanreotide

## Abstract

**Purpose:**

PREF-NET reported patients’ experience of Somatuline® (lanreotide) Autogel® (LAN) administration at home and in hospital among patients with gastroenteropancreatic neuroendocrine tumours (GEP-NETs).

**Methods:**

PREF-NET was a multicentre, cross-sectional study of UK adults (aged ≥ 18 years) with GEP-NETs receiving a stable dose of LAN, which comprised of (1) a quantitative online survey, and (2) qualitative semi-structured interviews conducted with a subgroup of survey respondents. The primary objective was the description of overall patient preference for home versus hospital administration of LAN. Secondary objectives included describing patient-reported opinions on the experience and associated preference for each administration setting, and the impact on healthcare utilisation, societal cost, activities of daily living and health-related quality of life (HRQoL).

**Results:**

In the primary analysis (80 patients; mean age 63.9 years), 98.7% (95% confidence interval [CI]: 96.1–100.0) of patients preferred to receive LAN at home, compared with 1.3% (95% CI: 0.0–3.9) who preferred the hospital setting. Among participants, over half (60.3%) received their injection from a non-healthcare professional. Most patients (79.5% [95% CI: 70.5–88.4]) reported a positive effect on HRQoL after the switch from hospital to home administration. Qualitative interviews (20 patients; mean age 63.6 years) highlighted that patients preferred home administration because it improved overall convenience; saved time and costs; made them feel more comfortable and relaxed, and less stressed; and increased confidence in their ability to self-manage their treatment.

**Conclusion:**

Almost all patients preferred to receive LAN treatment at home rather than in hospital with increased convenience and psychological benefits reported as key reasons for this preference.

**Supplementary Information:**

The online version contains supplementary material available at 10.1007/s00520-024-08377-7.

## Introduction

Gastroenteropancreatic neuroendocrine tumours (GEP-NETs) are a diverse and relatively rare group of neoplasms that arise from the diffuse neuroendocrine system [1]. Somatuline® (lanreotide) Autogel®/Depot (Ipsen) (herein referred to as LAN) is a long-acting somatostatin analogue indicated for the treatment of functional symptoms associated with NETs. Lanreotide is also indicated for anti-tumour treatment of grade 1 and a subset of grade 2 GEP-NETs (Ki67 index up to 10%) of midgut, pancreatic or unknown origin where the hindgut sites of origin have been excluded, in adults with unresectable locally advanced or metastatic disease [2].

LAN comes as a pre-filled, ready-to-use syringe and is administered every 28 days by deep subcutaneous injection [2]. In the UK, patients usually initiate LAN in the hospital setting, but once their dose is stable, they may transition to receive at-home injections. These at-home injections could be via a visiting healthcare professional (HCP), self-administration or a family member/friend (after appropriate training).

In a systematic literature review covering 13 studies, patients with cancer preferred to receive their anticancer drug injections at home rather than in hospital [3]. Hospital-based treatment can be stressful, disruptive and costly for patients and caregivers [3]. Similarly, in a literature review of at-home cancer treatment from 31 studies covering North and South America, Europe, Asia and Australia, 70% to 100% of patients preferred home administration [4]. In addition, over half of the patients reported improvements in well-being, activities of daily living, and family/social life, with benefits including convenience, comfort, reduced travel and financial burdens, limited waiting time, and greater ability to maintain daily family and social activities [4]. Both reviews highlighted the potential for at-home cancer treatment to reduce healthcare resource utilisation and infection exposure [3, 4]. Between study comparisons of several parameters such as HRQoL were hampered by the differing methodologies of the studies included in both reviews [3, 4].

Consistent with data for patients with other cancers, patients with NETs have reported appreciating the convenience of receiving at-home treatment, with benefits including increased independence and a reduced requirement for clinic visits [5, 6]. However, to date, there has been no evaluation of LAN administration setting preferences among patients with NETs in routine practice in the UK. PREF-NET aimed to generate real-world evidence describing the patient experience of LAN administration at home and in hospital, and the associated preference among patients with GEP-NETs in the UK. PREF-NET also assessed the impact of administration setting on activities of daily living, health-related quality of life (HRQoL), healthcare utilisation and societal cost.

## Methods

### Study design

PREF-NET was a cross-sectional, non-interventional, real-world study conducted at five centres in England and Wales between July 2021 and May 2022. Study sites were Liverpool University Hospitals NHS Foundation Trust, Cardiff and Vale University Health Board, University Hospitals Coventry & Warwickshire NHS Trust, University Hospitals Birmingham NHS Foundation Trust and Royal Free London NHS Foundation Trust.

Eligible patients had GEP-NETs and received LAN for tumour or symptom control in the home setting, but had prior experience in the hospital setting. The study had two parts: (1) a bespoke quantitative online patient outcomes survey, completed by the patient at a single point in time; and (2) semi-structured qualitative interviews conducted with a subgroup of patients who had completed the survey.

PREF-NET was performed in accordance with the Declaration of Helsinki and the Good Pharmacoepidemiology Practices from the International Society for Pharmacoepidemiology, and adhered to all applicable local regulatory requirements. The study protocol and supporting documents were submitted for National Health Service (NHS) Research Ethics Committee (REC) review via the Health Research Authority for the five UK sites. Research and development departments at participating sites issued capacity and capability statements, as confirmation of approval, before patient recruitment commenced (REC reference: 21/ES/0021; IRAS project ID: 286528).

### Participants

Inclusion criteria for participants included in the quantitative online survey were as follows:

- Aged ≥ 18 years
- Diagnosed with GEP-NETs
- Prescribed LAN and judged to be on a stable dose (120 mg for tumour control or > 2 injections at the same dose for symptom control)
- Had switched from hospital to home administration of LAN between 4 and 24 months before enrolment
- Willing and able to complete the study questionnaires

Participants included in the qualitative interviews were required to have switched from hospital to home administration relatively recently (between 4–12 months before enrolment) to reduce the risk of recall bias and were willing and able to complete the one-to-one interview and be audio-recorded.

Although anticipated recruitment was approximately 15–25 patients per site, sample size requirements were flexible to allow for oversampling from larger sites with access to more eligible patients should any site(s) under-recruit. Patients were invited on a first-come, first-served basis to participate in the qualitative interviews at the start of the online questionnaires. This selection occurred prior to the patient completing the questionnaire and without accessing the questionnaire data.

### Procedures and assessment

A mixed-methods study design was employed to collect quantitative data (via the online survey) on treatment administration setting preference and the overall patient experience, and qualitative data (via the one-to-one interviews) to provide deeper insights that could add to and help explain the quantitative findings.

The bespoke online survey was developed by the Patient Centred Outcomes team at OPEN VIE (London, UK), working with a clinical expert (the study Chief Investigator) to draft questions. Responses were collected via an established, secure, patient reported outcome-validated, electronic platform. All participants were recruited via UK NHS sites. Potentially eligible patients were identified by members of their care team during routine clinic, telephone or home contacts during the recruitment periods at each of the five sites (recruitment ran for approximately 4 months from the date of study initiation of the first hospital site).

Patients were free to complete the survey in their own time. The interviews were conducted on the telephone by an experienced qualitative interviewer from OPEN VIE, and on a different day to the online survey. A semi-structured guide was followed, but participants were free to direct the conversation to topics that they felt were relevant to their experience. The themes and sub-themes explored were focused on the positive and negative aspects of home and hospital administration of LAN, as well as the transition from hospital to home administration. Interviews were audio-recorded and transcribed into Word® (Microsoft, Redmond, WA). Data from these transcripts were entered into NVivo (QSR International, Burlington, MA) for coding.

The aim of the study was to generate real-world evidence to describe the patient experience of the administration of Somatuline® Autogel® in homecare and hospital settings. The primary endpoint from the online survey was the description of overall patient preference for home versus hospital administration of LAN. All study endpoints are listed in Table 1.

**Table 1:** Study endpoints

Endpoints	
Overall primary endpoint	
Description of overall patient preference for home versus hospital administration of LAN	
Secondary endpoints for the quantitative online survey	
Describe the demographics and clinical characteristics of patients	
Impact of the switch from hospital to home administration on:	• Overall injection experience (worsened; no change; improved) • Societal costs and activities of daily living • HRQoL (negative; no effect; positive) • Healthcare resource utilisation
Patient reports on how home administration compared with hospital administration, assessed using a 6-point scale (much worse; somewhat worse; about the same; somewhat better; much better; I do not wish to answer) for the following setting-related aspects:	• Time (travel, attending medical appointments) • Treatment-associated costs • Convenience of the treatment • Ability to be independent • Confidence in self-management of GEP-NETs • Ability to plan and/or go on holiday • Ability to engage in social activities • Relationships with family members/friends • Ability to work
Secondary endpoints for the qualitative interviews	
Patient experience of receiving LAN at home or in hospital, and the benefits and limitations of each setting	
Impact of administration setting on HRQoL, work productivity, and emotional and physical health	
Patient preference for administration setting (home or hospital) and the reasoning behind this choice	
Pre-specified subgroup analysis for the qualitative interviews: patients aged ≥ 65 years and < 65 years	
Subgroup analyses of key themes exploring the positive and negative aspects of home and hospital administration of LAN, as well as the transition from hospital to home treatment administration was stratified by patients aged ≥ 65 years and < 65 years to identify any differences between age subgroups	

*GEP-NET* gastroenteropancreatic neuroendocrine tumour, *LAN* lanreotide autogel, *HRQoL* health-related quality of life

### Statistical considerations

Because the aim of the study was to generate real-world evidence to describe the patient experience of administration of LAN in homecare and hospital settings, there were no hypotheses to be tested for the primary or secondary endpoints. No formal statistical testing was performed; analyses were primarily descriptive in nature, so no fixed sample size was required to derive conclusions. However, to ensure precision of the descriptive estimates, a target sample size of 80–90 participants was selected based on previous published research on LAN-treated patients [7–10]. For the qualitative interviews, a sample size of 15–20 participants was targeted; previous research has found that theme saturation (i.e. the point at which no new codes or themes are generated) is reached after around 12 interviews [11].

Descriptive statistics include the number of available data and missing data, as well as mean and standard deviation (SD) for quantitative variables and counts and percentages for categorical nominal variables. Percentages were based on the number of non-missing observations, and missing data were not replaced. Where appropriate, two-sided 95% confidence intervals (CIs) were generated. Data analyses were performed using SAS® (version 9.4 or higher; SAS Institute Inc., Cary, NC).

## Results

### Baseline characteristics

Eighty patients were enrolled and completed the online survey (Coventry, *n* = 24; Liverpool, *n* = 17; Cardiff, *n* = 16; Birmingham, *n* = 13; and London, *n* = 10). Overall, 52.0% (39/75) of patients were male and the mean ± SD age (*n* = 77) was 63.9 ± 10.6 years (Table 2). A total of 87.5% (70/80) of patients were receiving a 120 mg dose of LAN for tumour control. The majority (59.0%, *n* = 46) of patients had switched to home administration of LAN within the past year. Among them, seven (15.2%) patients switched to homecare owing to the impact of the COVID-19 pandemic. Among participants, LAN was administered by the patient (23.1%), their partner or family member (35.9%), a nurse (39.7%) or someone else (1.3%). After entering the survey portion of the study, twenty patients were then included in the analysis of qualitative interviews; not all of these patients had completed every survey question. Baseline characteristics for these patients were similar to those of the full survey population (Table 2).

**Table 2:** Patient characteristics

Variable	Category	Survey population (*N* = 80)^a^	Interview population (*n* = 20)
Age, years, mean (SD)	–	63.9 (10.6)	63.6 (8.7)
Sex, male, n (%)	–	39 (52.0)	12 (60.0)
Time from diagnosis to initial injection (months)^b^, mean (SD)		11.2 (29.6)	5.8 (9.4)
Time since diagnosis (months)^c^, mean (SD), median		33.7 (53.3) 17.9	18.8 (17.1) 12.8
Time since switch to home administration, *n* (%) [95% CI]	< 6 months	23 (29.5)	6 (30.0)
	6–12 months	23 (29.5)	9 (45.0)
	> 12 months	32 (41.0)	5 (25.0)
Switch to homecare due to COVID-19,^d^ *n* (%) [95% CI]	Yes	7 (15.2) [4.8–25.6]	5 (33.3) [9.5–57.2]
	No	39 (84.8) [74.4–95.2]	10 (66.7) [42.8–90.5]
Administration of medication, *n* (%) [95% CI]	Myself	18 (23.1) [13.7–32.4]	6 (30.0) [9.9–50.1]
	Partner/family member	28 (35.9) [25.3–46.5)	9 (45.0) [23.2–66.8]
	Nurse	31 (39.7) [28.9–50.6]	5 (25.0) [6.0–44.0]
	Someone else	1 (1.3) [0.0–3.8]	0 (0) [0.0–0.0]

^a^The numbers of respondents were 77 (age), 75 (sex), 65 (time from diagnosis to initial injection), 69 (time since diagnosis), 46 (switch to homecare due to COVID-19) and 78 (time since switch to home administration, administration of medication), respectively

^b^Variables were created from injection start date and diagnosis date

^c^Variables were created from date of diagnosis to date of survey completion

^d^As the first inclusion occurred in July 2021, more than 1 year after the start of the COVID-19 pandemic, this question was probably applicable to all patients, but was asked only to patients switching within 1 year of it

CI, confidence interval; SD, standard deviation

### Overall patient preference

**Fig. 1:**
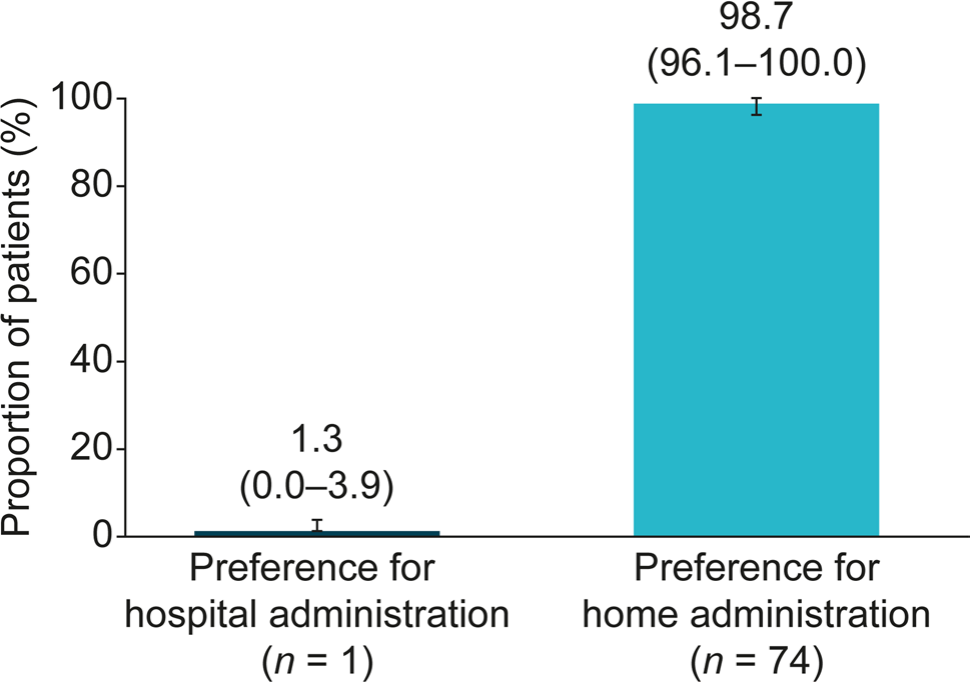
Preference for LAN administration setting**.** Survey population (*N* = 80 with five missing values). Whiskers represent 95% confidence intervals. LAN, lanreotide autogel

In the primary analysis (75 respondents), 74 (98.7% [95% CI: 96.1–100.0]) patients preferred to receive LAN at home, compared with one (1.3% [95% CI: 0.0–3.9]) who preferred the hospital setting (Fig. 1). Data from five patients was missing from the analysis, three patients could not be assigned to a subgroup (two patients who had not completed the survey and one who did not specify the mode of LAN administration) and two patients did not finish completing the survey. Preference for home administration was similar between patients who administered LAN themselves or were injected by their partner or family member (97.7% [95% CI: 93.3–100.0]) versus those who were administered LAN by a visiting HCP (100% [95% CI: 100.0–100.0]).

### Overall injection experience

Among 76 respondents who rated their overall injection site experience with home versus hospital administration, 64 (84.2% [95% CI: 76.0–92.4]) reported improvement, 11 (14.5% [95% CI: 6.6–22.4]) reported no change, and one (1.3% [95% CI: 0.0–3.9]) reported that it had worsened. The 64 patients who reported improvement were evenly distributed between the treatment groups: 38 of 46 (86.4%) who administered LAN themselves or received injections from their partner or family member, and 25 of 31 (80.6%) who received LAN injections from an HCP (in addition to one patient who reported receiving the injection from ‘someone else’).

### Injection experience and activities of daily living

**Fig. 2:**
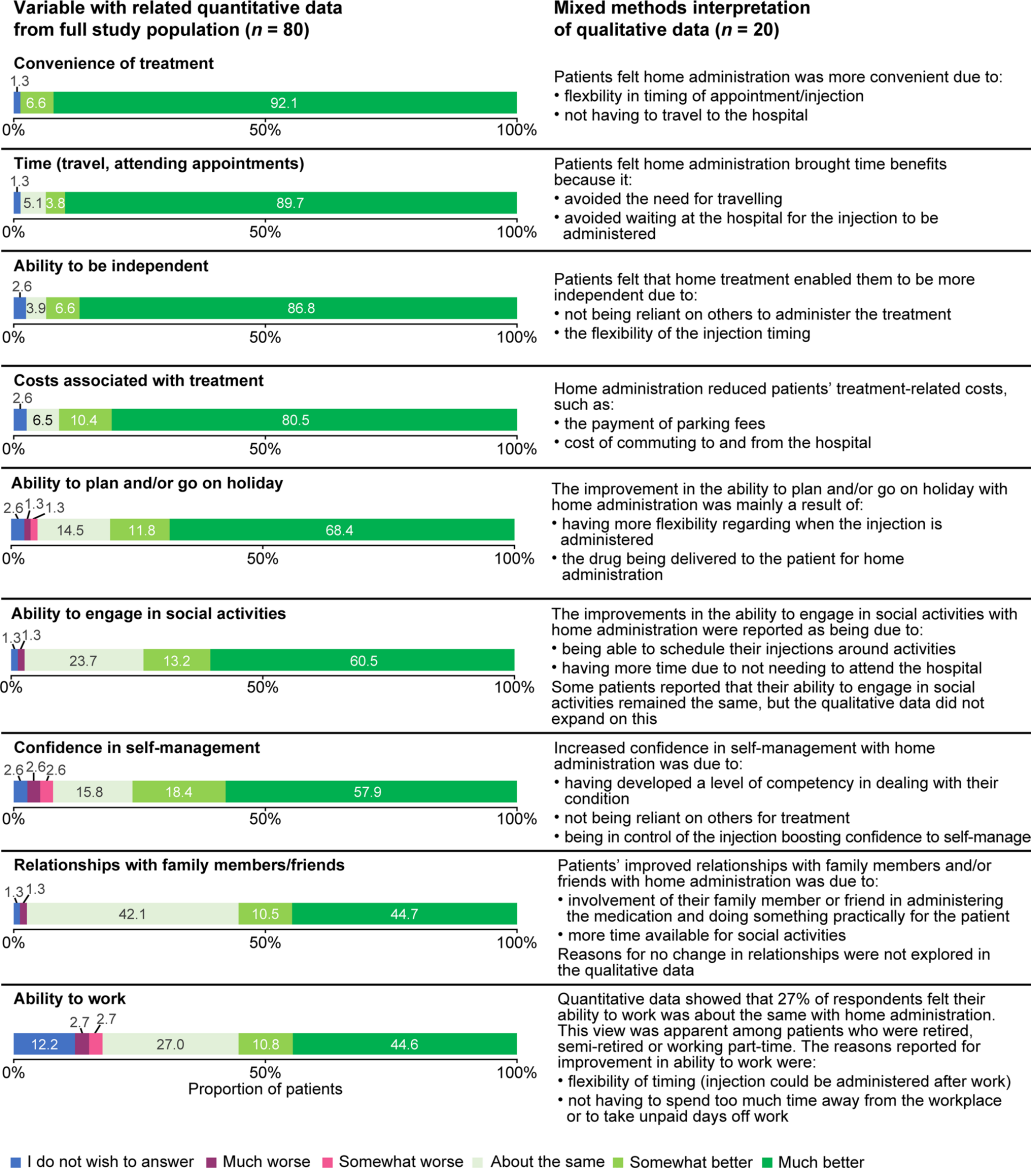
Impact on activities of daily living with LAN administration at home versus hospital: integrated results from survey and interview populations**.** LAN, lanreotide autogel

For all nine fields examined, the majority of patients reported that home administration was ‘much better’ or ‘somewhat better’ than hospital administration for activities of daily living (Fig. 2); particularly with regard to the convenience of treatment (*n* = 75/76; 98.7%), time savings (travel and attending medical appointments; *n* = 73/78; 93.6%) and the ability to be independent (*n* = 71/76; 93.4%).

The majority of patients also considered home administration to be better than hospital administration with regard to treatment-associated costs (*n* = 70/77; 90.9%), confidence in self-management (*n* = 58/76; 76.3%), relationships with family and friends (*n* = 42/76; 55.3%), and the ability to work (*n* = 41/74; 55.4%), go on holiday (*n* = 61/76; 80.3%) and engage in social activities (*n* = 56/76; 73.7%). Proportionately more patients using self/partner/family member versus visiting HCP administration reported that their confidence in self-management after switching to the home setting was ‘much better’ (68.9% versus 43.3%) or ‘somewhat better’ (22.2% versus 13.3%). Hospital administration also had an impact on the time that patients had available to do daily activities. Among 78 patients, 34.6% (*n* = 27) found that the time required for hospital administration caused them to miss paid work. Additional activities missed owing to the time required for hospital visits were leisure activities (23.1%), housework (19.2%), volunteering (1.3%) and other (21.8%) (Supplementary Table 1).

### HRQoL

**Fig. 3:**
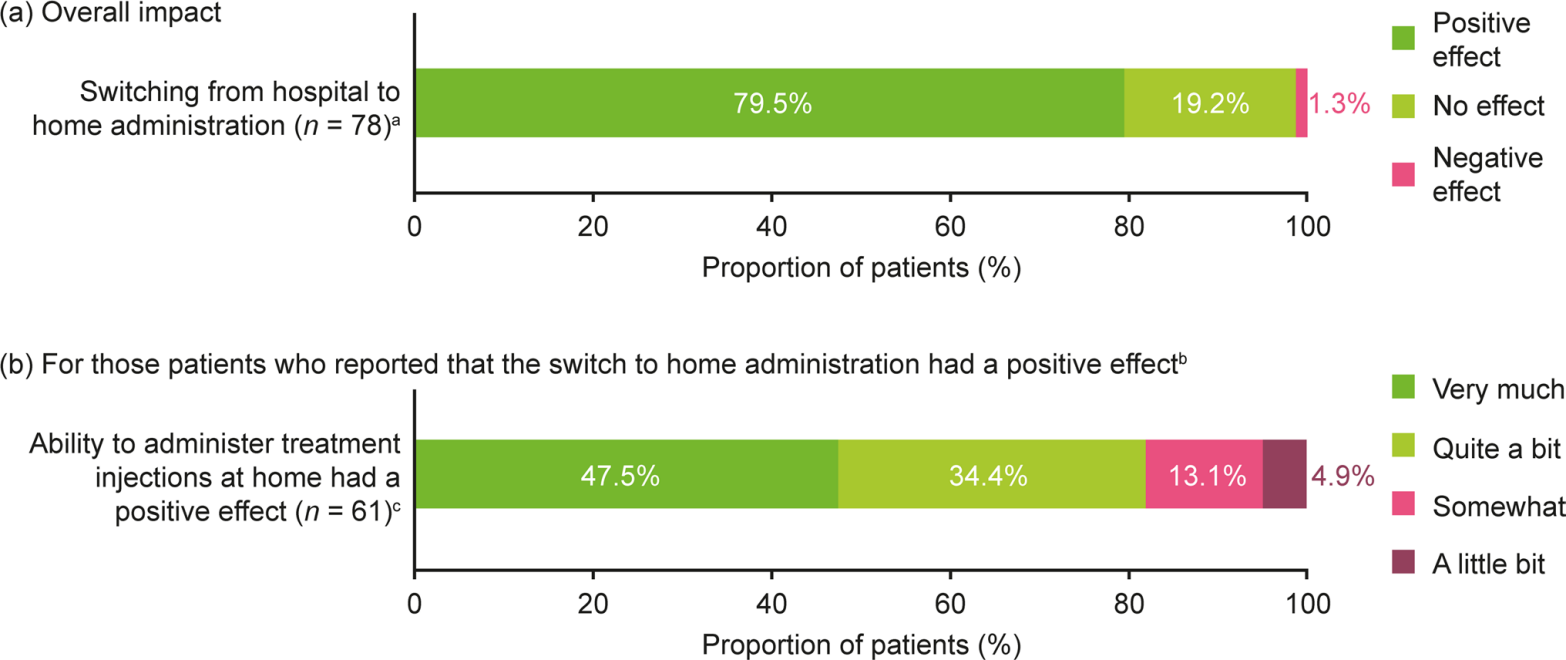
Impact on HRQoL of switching from hospital to home administration. ^a^Survey population (*N* = 80 with two missing values). ^b^The patient who reported a negative effect in part A indicated that the ability to administer treatment injections at home had ‘quite a bit’ of a negative effect. ^c^One missing value. HRQoL, health-related quality of life

Of 78 respondents, 62 (79.5% [95% CI: 70.5–88.4]) reported a positive effect on their HRQoL of switching from hospital to home administration, 15 (19.2% [95% CI: 10.5–28.0]) reported no effect, and one (1.3% [95% CI: 0.0–3.8]) reported a negative effect (Fig. 3A). Among 60 of these 61 respondents with available data, 29 (47.5% [95% CI: 35.0–60.1]) indicated that the positive effect was ‘very much’ and 21 (34.4% [95% CI: 22.5–46.4]) indicated ‘quite a bit’ (Fig. 3B).

### Healthcare resource use: Impact of treatment setting switch

Of 77 respondents, 69 (89.6% [95% CI: 82.8–96.4]) reported that the number of hospital or clinic visits was reduced with home administration. In the 4 months prior to data collection, the mean (SD) number of times that respondents (*n* = 73) had contact with a hospital or clinic was 1.5 (2.0), and of the respondents (*n* = 42) that had a face-to-face appointment, the mean (SD) number of times these occurred was 2.3 (1.9).

### Interpretation of patient quotes by theme from the qualitative interview responses

The qualitative interviews provided more in-depth information on why home administration was preferred by most participants (Fig. 2 and Table 3). Patients highlighted that home injection avoided the need to travel to the hospital and wait for their appointment – thereby saving time and costs (fuel, transportation and parking) – and reported that the increased flexibility improved overall convenience. In particular, time savings made it easier to work, take holidays and engage in social activities.

**Table 3:** Interpretation of patient quotes on the key sub-themes in qualitative interview responses

Theme	Key sub-themes apparent in responses	Interpretation of patient quotes
Perceived benefits of home administration	Choice and flexibility of injection timing	Patients noted the choice and flexibility of injection timing with home administration and discussed the freedom and convenience of this in terms of planning their injections to fit their daily lives
	Avoiding exposure to COVID-19	The reduced risk of exposure to COVID-19 with home administration was a benefit of home administration to some patients
	Time saving	Patients felt there was more time saved with home administration than hospital administration, because patients did not need to travel to and wait for their appointment
	Comfort of the home setting	The comfort of the home setting provided patients with a sense of familiarity that they did not experience with the hospital setting, allowing for a more personal approach
	More confidence to self-manage condition	Patients felt home administration instilled confidence in their management of their condition, offering a level of empowerment
	Certainty of receiving treatment	Patients indicated that home administration offered a level of certainty that they would receive their treatment and not miss doses through the cancellation of hospital appointments
	Freeing up healthcare resources	Patients noted the realisation that home administration reduces the workload of HCPs, ultimately saving the NHS money
Positive impacts of home administration	Quality of life: psychological	Home administration had a positive impact on patients’ psychological QoL, as it was more relaxing and took away the stress of travelling to and from the hospital
	Quality of life: social life and relationships	Home administration had a positive impact on patients’ QoL in terms of their social lives and relationships
	Work	Patients felt there was a positive impact on work with home administration, as injections could be planned around their work schedule and less time off work was required
	Financial	The financial savings were a positive impact of home administration, as patients could avoid costs such as parking and fuel, which are associated with hospital administration
Perceived limitations of home administration	Less interaction and communication with HCPs	Patients noted that they had less interaction and communication with their HCPs with home administration
	Challenges of drug administration	Patients referred to the challenges of drug administration when administering at home
	Cold chain challenges	Concerns surrounding the storage of the injections was seen as a limitation of home administration, concerns centred around power cuts and storage in a domestic refrigerator
	Inefficient and inconvenient drug delivery	Patients noted issues with the delivery of their drug as a limitation of home administration, which in some cases caused delays in the administration of their next dose
Perceived benefits of hospital administration	Feelings of safety	One patient discussed how they felt the hospital environment was a safe option because if anything goes wrong with the treatment, HCPs are on hand to help
	Better communication opportunities with HCPs	Patients had opportunities to discuss concerns with HCPs around hospital administration, including the opportunity to ask any questions about their disease and treatment
Perceived limitations of hospital administration	Long commute to the hospital	Patients noted that the often long commute to the hospital for their injection appointments was a limitation of hospital administration
	Long waiting time while at the hospital	Patients noted that the often long waiting times while at the hospital was a limitation of hospital administration
	Inconvenient appointment times	Patients noted that inconvenient appointment times were a limitation of hospital administration
Negative impacts of hospital administration	Quality of life: physical	Patients noted the waiting and travel associated with hospital appointments had a negative impact on their physical health/well-being
	Quality of life: psychological	Patients noted the negative psychological impact of hospital administration, due to both the commute and hospital environment
	Quality of life: social life and relationships	Patients referred to how hospital administration interfered with their interactions with friends and family, negatively impacting their social lives and relationships
	Work	Patients noted the negative impact hospital administration had on their work life, primarily due to the time off needed to attend appointments
	Financial	Patients felt there was a financial burden associated with hospital administration, due to the costs associated with travelling to the appointment and missing work
Transition from hospital to home administration	COVID-19 as a reason for switch	Some patients noted that their primary reason for switching to home administration was the COVID-19 pandemic
	Role of HCPs in switch	Patients referred to the role of their HCP in the switch to home administration, particularly their provision of guidance on self-injection
	Negative feelings around switching to home administration	Negative feelings surrounding the switch related to concerns the effect home administration would have on relationships and a lack of confidence
	Positive feelings around switching to home administration	Patients referred to the confidence instilled in them by their HCPs, which made them feel positive about the switch to home administration

HCP, healthcare professional; QoL, quality of life; NHS, National Health Service

From a psychological perspective, patients preferred home administration because they felt more comfortable, relaxed and less stressed than in hospital, and because it helped them to worry less about the seriousness of their illness and to not think about it as much. Patients also highlighted an increased confidence in their ability to self-manage their treatment when receiving home administration, which was associated with feeling more in control of their disease; this was particularly apparent among individuals whose LAN injection was self- or partner administered.

The interviews also highlighted potential limitations of home administration compared with hospital-based care, such as reduced interaction with HCPs, challenges with drug storage and administration, and inefficient and inconvenient home delivery of drugs.

### Impact of patient age: subgroup analyses

Subgroup analyses identified no major differences between patients aged 65 years or older versus those aged less than 65 years for the themes and sub-themes that explored the positive and negative aspects of home and hospital administration of LAN, as well as the transition from hospital to home settings (Supplementary Table 2).

## Discussion

PREF-NET was the first study to evaluate patients’ experiences of LAN administration in home and hospital settings in routine clinical practice in the UK.

Almost all patients (98.7%) preferred to receive LAN at home rather than in hospital, with 84.2% of patients indicating that their overall injection experience was improved with home versus hospital administration. Importantly, the administration setting itself appeared to be the key factor associated with the preference because there was no significant difference in preference between patients who administered LAN by themselves or via their partner or family member versus those who received LAN injections from an HCP. It is notable that over half of those receiving LAN at home had their injections administered by a non-HCP (i.e. a family member or friend). Over three-quarters of patients (79.5%) reported a positive impact on their HRQoL when switching from hospital to home administration, with almost half of these patients (47.5%) indicating that the extent of this effect was ‘very much’. Patients' preference for at-home administration may be, in part, supported by having LAN as a ready-to-use pre-filled syringe. Indeed, patients have previously reported that they were less likely to experience prolonged pain or technical issues with this device than with the latest octreotide long-acting release formulation (Novartis) [12]. Additionally, in an international simulated-use study, nurses administering lanreotide strongly preferred the user experience of the LAN syringe used in this study (Somatuline Autogel) versus an alternative, the Lanreotide Pharmathen syringe [13]. A further consideration is that the level of information provided to patients with NETs by HCPs may influence the decisions to offer and undertake home administration. Indeed, in a recent French study, high scores were reported for patient perceptions of the level of information provided when initiating LAN treatment for GEP-NETs [14].

Overall, most patients reported that the impact on activities of daily living was better with home versus hospital administration. In particular, convenience of treatment (98.7%), time travelling to and attendance at medical appointments (93.5%) and the ability to be independent (93.4%) was better with home (versus hospital) administration. Treatment-associated costs (90.9%), confidence in self-management (76.3%), relationships with family and friends (55.3%), and the ability to work (55.4%), go on holiday (80.3%) and engage in social activities (73.7%) were also reported by most patients to be better with home (versus hospital) administration.

Patients reported benefits of home administration on healthcare utilisation, with 89.6% of patients reporting a reduction in their hospital and clinic visits. Although these findings were based on patient reports and not on direct evaluation of resource use, they do align with data from a previous UK study of unreimbursed costs associated with transition to home administration of LAN for patients with GEP-NETs [15]. In the previous UK study, 50% uptake in the use of LAN home administration had the potential to reduce unreimbursed costs to the NHS by £11,178,960 over a 5-year period [15].

As well as cost considerations, staff resourcing is a significant challenge during this time of great pressures on the UK NHS. Utilising home-based LAN administration may reduce staff resourcing requirements for managing patients with NETs and provide a cost-effective alternative to hospital-based administration for delivering high-quality patient care [16]. Although it must be acknowledged that some patients may not be suitable for home administration, the overwhelming preference for home administration reported here provides strong justification for promoting further uptake of home-based LAN administration. Indeed, with no observed differences between patients aged ≥ 65 years and < 65 years in the sub-analyses of PREF-NET, use of home-based LAN could be promoted to patients of all ages. Expansion of self- or partner-administration of LAN (versus HCP home visits) would be expected to have an even greater positive impact on healthcare resource needs. The hospital at home concept is expanding across a wide range of therapy areas and has been shown to be beneficial from an economic standpoint and for controlling the flow of patients in hospitals [17].

The qualitative interviews included in PREF-NET provided deeper insights into why home administration was preferred by most participants and highlighted the negatives/limitations of hospital administration. Patients appreciated avoiding the need to travel and wait for appointments because this freed up their time and avoided costs associated with fuel, transportation, and parking. Patients also reported feeling more comfortable, relaxed and less stressed than in the hospital environment. It should be noted that recruitment into PREF-NET occurred during the COVID-19 pandemic. The impact of the pandemic was not specifically assessed, but this time frame may have contributed to patients’ preference for avoiding the hospital setting because of their desire to minimise their risk of infection. However, only a small number of patients (15.2%) indicated that they switched to homecare because of COVID-19, so the impact on the overall study findings is likely to be limited. Encouragingly, patients described having increased confidence in their ability to self-manage their treatment when receiving LAN at home, particularly among those whose LAN was self- or partner-administered.

Although the overall findings of PREF-NET were overwhelmingly supportive of home administration, patients did report some negative aspects. These included reduced interaction with HCPs, challenges of drug administration and storage, and inconvenience of arranging drug deliveries. Another potential disadvantage of transitioning from hospital to home administration rather than to general practitioner (GP)-led administration could be the impact on patients’ relationships with their GP medical practice. Reduced interaction with the medical practice may lead to reduced patient confidence in their GP’s awareness of their condition.

Potential ways to mitigate against concerns regarding lack of HCP contact are to ensure patients who have switched to home administration are aware of the importance of still maintaining regular contact with the hospital NET service. It is worth noting that home administration of LAN in PREF-NET was managed by highly specialised NET service staff, who provided information/support. Optimising the efficiency of drug deliveries would also improve the experience for patients receiving LAN at home.

Key strengths of PREF-NET include the bespoke nature of the online survey, the broad inclusion criteria and the fact that data reflect the experience of patients receiving care in standard clinical practice. The baseline characteristics of patients included in PREF-NET were similar to those of at least three other key studies assessing LAN treatment in patients with NETs [18–20], indicating that the PREF-NET population was reflective of the general population of patients receiving LAN. The inclusion of data on negative aspects of each treatment setting was another study strength.

Recall bias is a limitation of this type of study design, particularly given that some patients switched from hospital administration up to 24 months prior to inclusion in the study. The approach of healthcare providers at each study site inviting patients to participate may have contributed to selection bias; however, because most patients at the five participating centres are routinely transferred to home administration, the study population was considered representative of patients with NETs in England and Wales. However, patients such as those with learning difficulties who were not able to complete the survey themselves were excluded. The broad inclusion criteria were designed to minimise the impact of selection bias, as was the method of recruitment of patients to the qualitative interviews. Nonetheless, some patients do not receive home administration, instead receiving injections at their GP or in secondary care; these patients are not represented in the current study population. It is also important to remember that these data are specific to England and Wales and may not translate to other healthcare systems. This is particularly true for countries where, unlike the NHS system, treatment is not free at the point of care. However, a recent study in four European countries showed a high level of patient satisfaction with patient support programmes that facilitated LAN treatment at home (predominantly via HCP-administration), suggesting that the learnings from PREF-NET may indeed be relevant, at least in part, to other healthcare systems [21]. Finally, PREF-NET did not assess whether the preference for home administration was linked to any improvements in treatment adherence and/or patient outcomes for those receiving LAN at home versus in hospital; this would be an interesting topic for future research.

In conclusion, PREF-NET was the first study to assess patients’ preference of LAN administration setting in routine practice in England and Wales. The findings demonstrated that almost all patients (98.7%) preferred to receive LAN treatment at home, compared with in hospital, and more than three-quarters of patients (79.5%) reported a positive impact on their HRQoL when switching from hospital to home administration. Convenience and the psychological benefits of receiving treatment at home were reported as key reasons for this preference. Overall, these data indicate that the decision to receive LAN at home is a valued treatment option for patients with GEP-NETs regardless of their age.

### Supplementary Information

Below is the link to the electronic supplementary material.

Supplementary file1 (DOCX 293 KB)

## Data Availability

Qualified researchers may request access to patient-level study data that underlie the results reported in this publication. Additional relevant study documents, including the clinical study report, study protocol with any amendments, annotated case report form, statistical analysis plan and data set specifications may also be made available. Patient-level data will be anonymised, and study documents will be redacted to protect the privacy of study participants. Where applicable, data from eligible studies are available 6 months after the studied medicine and indication have been approved in the US and EU or after the primary manuscript describing the results has been accepted for publication, whichever is later. Further details on Ipsen's sharing criteria, eligible studies and process for sharing are available here (https://vivli.org/members/ourmembers/). Any requests should be submitted to http://www.vivli.org for assessment by an independent scientific review board.

## Declarations

### Medical writing support

The authors thank Kirsty Walters (PhD) and Tamzin Gristwood (PhD) of Oxford PharmaGenesis, Oxford, UK, who provided medical writing and editorial support, which was sponsored by Ipsen in accordance with Good Publication Practice (GPP 2022) guidelines.
