# Supplementary material for: PREF-NET: a patient preference and experience study of lanreotide autogel administered in the home versus hospital setting among patients with gastroenteropancreatic neuroendocrine tumours in the UK
Source: Support Care Cancer. 2024 Feb 29;32(3):199. doi: 10.1007/s00520-024-08377-7 (PMC10904552; doi:10.1007/s00520-024-08377-7)
Supplement: Supplementary file 1 — Supplementary file1 (DOCX 293 KB) [file 520_2024_8377_MOESM1_ESM.docx]

**PREF-NET: a patient preference and experience study of** **lanreotide autogel administered in**

**the home versus hospital setting among patients with** **gastroenteropancreatic neuroendocrine**

**tumours in the UK**

Mohid S Khan,^1*^ Kathryn Cook,^1^ Martin O Weickert,^2^ Louise Davies,^2^ D Mark Pritchard,^3^ Melissa Day,^3^ Tahir Shah,^4^ Diana Hull,^4^ Martyn Caplin,^5^ Melissa Back,^5^ Christelle Pommie,^6^ Kate Higgs^7^

^1^Cardiff and Vale University Health Board, Cardiff, UK

^2^The ARDEN NET Centre, ENETS Centre of Excellence, University Hospitals Coventry & Warwickshire NHS Trust, Coventry, UK

^3^Liverpool University Hospitals NHS Foundation Trust, Liverpool, UK

^4^University Hospitals Birmingham NHS Foundation Trust, Birmingham, UK

^5^Royal Free London NHS Foundation Trust, London, UK

^6^Ipsen, Boulogne-Billancourt, France

^7^Ipsen, Slough, UK

*Corresponding author: Mohid S Khan

Email: [KhanMS14@cardiff.ac.uk](mailto:KhanMS14@cardiff.ac.uk)

**Target journal:** *Supportive Care in Cancer*

**Short title:** PREF-NET: patients’ preference of LAN administration setting

**Plain language summary**

Drugs called somatostatin analogues (SSAs) are commonly accepted first-line treatments for people living with gastroenteropancreatic neuroendocrine tumours (GEP-NETs). SSAs are given as regular injections and can be used for many years. Somatuline® (lanreotide) Autogel® (referred to as LAN) is a type of SSA. When people first start receiving LAN in the UK, they are usually given the injection in hospital. However, once people are receiving a stable dose of LAN they can receive their injections at home. Either the person being treated (or a nominated adult, such as a family member) can be trained to inject LAN at home or the injection can be done by a healthcare professional at a hospital, medical practice or in the person’s home. In this study, called PREF-NET, we used an online survey to ask 80 people with GEP-NETs about their experience with LAN injections at home and in hospital in the UK. Twenty of these people were also interviewed to get more information about their experience. We found that nearly all the people who answered the survey (74 out of 75; 98.7%) preferred to receive their LAN treatment at home instead of in hospital. Most people (64 out of 76; 84.2%) said that their overall experience of LAN injections was better when they had their injections at home instead of in hospital. People told us that they preferred the home over the hospital setting for LAN injections because it was more convenient and saved them time and money. It also made people feel more comfortable and less stressed than going to the hospital for their LAN injection, and made people feel more confident in their ability to manage their illness. The results from our study show that it is important to give people with GEP-NETs the chance to be injected with LAN at home instead of in hospital.

**Graphical abstract**

**
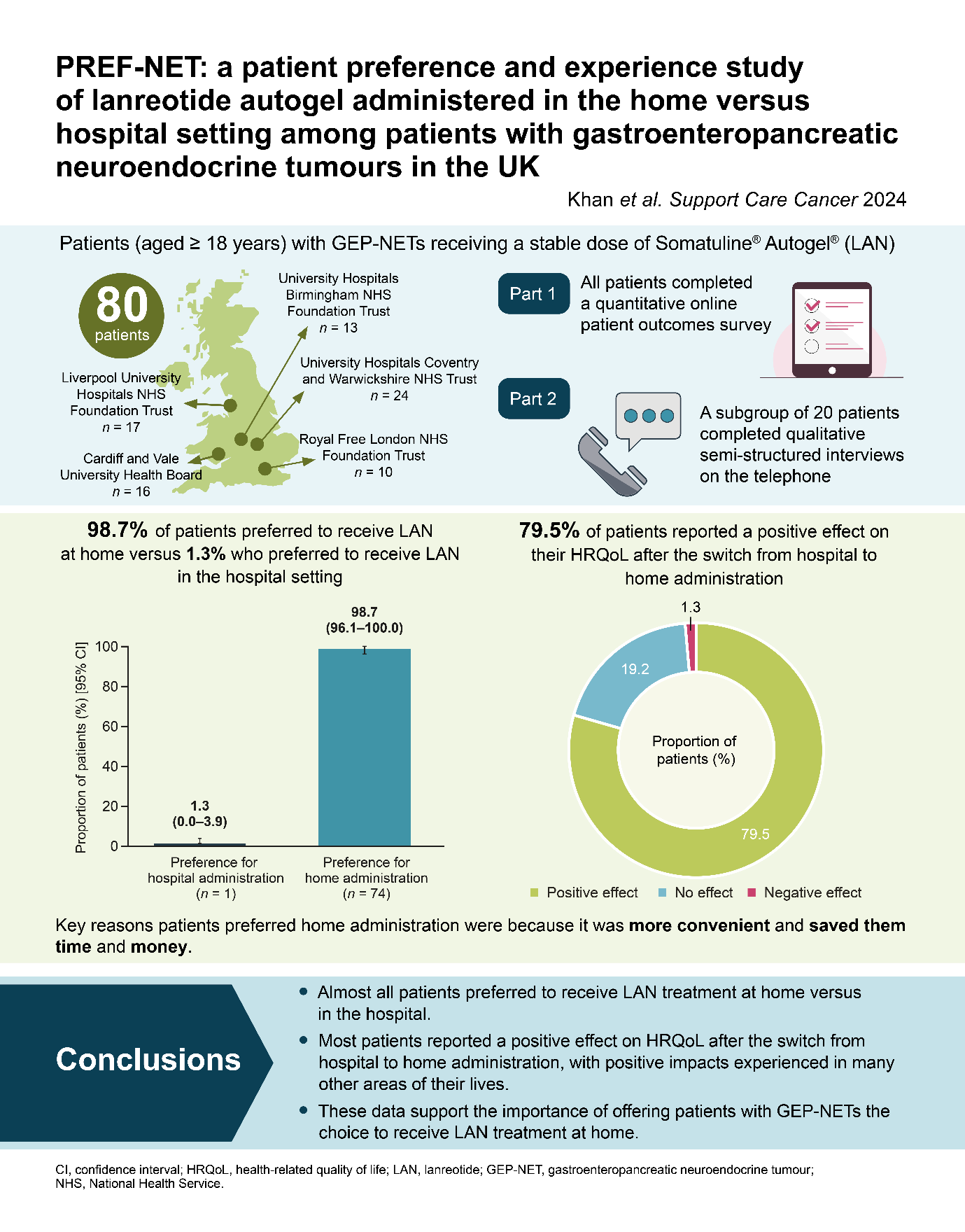
**

**SUPPLEMENTARY TABLE 1** Impact of treatment administration setting on costs and patient time to do daily activities

| **Variable** | **Category** | **Survey population  (*N* = 80)** | **Self/partner/family member administration^a^**  **(*n* = 46)** | **HCP administration^a^**  **(*n* = 31)** |
| --- | --- | --- | --- | --- |
| Distance from hospital/clinic (miles), mean (SD) | –  Missing values | 23.4 (27.5)  6 | 25.8 (24.3)  3 | 20.5 (31.9)  1 |
| Main mode of transportation, *n* (%) [95% CI] | Car  Train  Bus  Taxi  Walking/bicycle  Non-emergency NHS-funded patient transport  Volunteer transport  Missing values | 67 (85.9) [78.2–93.6]  4 (5.1) [0.0–10.0]  1 (1.3) [0.0–3.8]  1 (1.3) [0.0–3.8]  2 (2.6) [0.0–6.1]  3 (3.8) [0.0–8.1]  0 (0.0) [0.0–0.0]  2 | 38 (82.6) [71.7–93.6]  4 (8.7) [0.6–16.8]  1 (2.2) [0.0–6.4]  1 (2.2) [0.0–6.4]  2 (4.3) [0.0–10.2]  0 (0.0) [0.0–0.0]  0 (0.0) [0.0–0.0]  0 | 28 (90.3) [79.9–100.0]  0 (0.0) [0.0–0.0]  0 (0.0) [0.0–0.0]  0 (0.0) [0.0–0.0]  0 (0.0) [0.0–0.0]  3 (9.7) [0.0–20.1]  0 (0.0) [0.0–0.0]  0 |
| Total cost of a visit (including outward and return journey, transportation (patient and accompanying person) and parking (£), mean (SD) | –  Missing values | 6.6 (14.3)  11 | 9.0 (18.1)  5 | 3.1 (2.5)  4 |
| Duration of travel time (one way, mins), mean (SD) | –  Missing values | 48.8 (50.8)  4 | 50.1 (35.8)  0 | 47.4 (69.5)  2 |
| Duration of visit (mins), mean (SD) | –  Missing values | 51.9 (30.3)  4 | 55.7 (35.1)  0 | 47.1 (19.4)  2 |
| Activities missed due to visit,  *n* (%) [95% CI] | Housework  Leisure activities  Paid work  Volunteering  Other  Missing values | 15 (19.2) [10.5–28.0]  18 (23.1) [13.7–32.4]  27 (34.6) [24.1–45.2]  1 (1.3) [0.0–3.8]  17 (21.8) [12.6–31.0]  2 | 7 (15.2) [4.8–25.6]  13 (28.3) [15.3–41.3]  19 (41.3) [27.1–55.5]  0 (0.0) [0.0–0.0]  7 (15.2) [4.8–25.6]  0 | 8 (25.8) [10.4–41.2]  5 (16.1) [3.2–29.1]  8 (25.8) [10.4–41.2]  1 (3.2) [0.0–9.5]  9 (29.0) [13.1–45.0]  0 |

^a^Three patients could not be assigned to a subgroup: two patients did not complete the survey and one patient answered, ‘Someone else’ to the question ‘Who administers your Somatuline Autogel injections at home’.

CI, confidence interval; SD, standard deviation.

**SUPPLEMENTARY TABLE 2** Subgroup analysis by age on the interpretation of patient quotes on the key sub-themes in qualitative interview responses

| **Theme** | **Key sub-themes apparent in responses** | **</≥65 years subgroup analysis of interpretation of patient quotes (difference or not between subgroups)** |
| --- | --- | --- |
| **Perceived benefits of home administration** | Choice and flexibility of injection timing | No difference. |
|  | Avoiding exposure to COVID-19 | Both age groups referred to avoiding exposure to COVID-19, however more patients in the ≥65 years category referred to this benefit, suggesting that they were more aware of the risk of catching COVID-19 and the importance of remaining at home or within their ‘bubble’ in order to avoid exposure. |
|  | Time saving | No difference. |
|  | Comfort of the home setting | No difference. |
|  | More confidence to self-manage condition | No difference. |
|  | Certainty of receiving treatment | No difference. |
|  | Freeing up healthcare resources | No difference. |
| **Positive impacts of home administration** | Quality of life: psychological | No major difference was observed between the subgroups, however some patients in the <65 years group referred to how home administration made them feel less ill. |
|  | Quality of life: social life and relationships | No major difference was observed between the subgroups, however those in the ≥65 years group made specific reference to an improved relationship with the partner administering their injection. |
|  | Work | Although similar numbers in both age groups referred to how home administration positively impacted their work life, more patients <65 years identified this sub-theme, likely due to the fact there were more in this age group who were still working. |
|  | Financial | No difference. |
| **Perceived limitations of home administration** | Less interaction and communication with HCPs | No difference. |
|  | Challenges of drug administration | Although both age groups referred to some of the issues associated with drug administration at home. Those <65 years described physical limitations that interfered with self-administration whereas those ≥65 years talked about the negative psychological impact experienced by their family member who was administering the injection. |
|  | Cold chain challenges | Both age groups discussed the challenges of storing the injections, but those <65 years referred to the effect power cuts may have on drug storage compared with those ≥65 years who had concerns surrounding whether their domestic refrigerator was the correct temperature for storage. |
|  | Inefficient and inconvenient drug delivery | No difference. |
| **Perceived benefits of hospital administration** | Feelings of safety | Only one patient <65 years noted increased feelings of safety with hospital administration vs home administration. |
|  | Better communication opportunities with HCPs | Both age groups noted the better communication opportunities with hospital administration, however more patients in the <65 years category referred to this benefit, indicating communication was more important in the younger age group. |
| **Perceived limitations of hospital administration** | Long commute to the hospital | No difference. |
|  | Long waiting time while at the hospital | No difference. |
|  | Inconvenient appointment times | No difference. |
| **Negative impacts of hospital administration** | Quality of life: physical | Both age groups identified that hospital administration had a negative impact on their physical well-being, however more patients referred to this concept among those aged <65 years, suggesting this impact was more important in this age group. |
|  | Quality of life: psychological | Although both age groups referred to the psychological impact of hospital administration, more patients in the ≥65 years category referred to it, suggesting a potentially greater impact on psychological well-being in these patients. |
|  | Quality of life: social life and relationships | No difference. |
|  | Work | Both age groups discussed the negative impact of hospital administration on work, however more patients in <65 years group made references to this sub-theme; reflective of the fact more individuals in this age group were part of the workforce. |
|  | Financial | Both age groups identified the negative impact of hospital administration on finances, however more patients ≥65 years made references within this sub-theme. |
| **Transition from hospital to home administration** | COVID-19 as a reason for switch | No difference. |
|  | Role of HCPs in switch | No difference. |
|  | Negative feelings around switching to home administration | Both age groups discussed negative feelings, however, the reason for the negative feelings differed slightly. Those <65 years described feeling fearful that the partner administration at home would influence their relationship, whereas those ≥65 years reported on a lack of confidence in having to self-inject. |
|  | Positive feelings around switching to home administration | No difference. |

HCP, healthcare professional.
